# Pancreatic sphincterotomy allows removal of a fractured stone basket trapped in the pancreatic duct after lithotripsy

**DOI:** 10.1055/a-2134-8947

**Published:** 2023-08-30

**Authors:** Yan Chen, Li Yang, Ting Yang, Cui Liu, Jie Chen

**Affiliations:** 1 Digestive Endoscopy Center, Changhai Hospital, Shanghai, China; 2 Department of Gastroenterology, Changhai Hospital, Shanghai, China

A 57-year-old woman presented with recurrent epigastric pain. Computed tomography of the abdomen revealed a 26-mm pancreatic stone obstructing the main pancreatic duct (MPD) in the body portion ( Fig. 1 ). We performed three sessions of extracorporeal shockwave lithotripsy (ESWL) using a third-generation lithotripter (Delta Compact II; Dornier MedTech, Weßling, Germany). Up to 5000 shocks were delivered per therapeutic session on a scale of 1 to 6, with a frequency of 120 shocks/min. Endoscopic retrograde cholangiopancreatography (ERCP) was routinely performed to remove the pancreatolith after ESWL to avoid steinstrasse. After sphincterotomy, we attempted to remove the pancreatolith with an eight-wire basket (MB5-2X4-8; Wilson-Cook Medical Inc., Bloomington, Indiana, USA); however, the basket became trapped by the calculi near the pancreatic orifice at the papilla ( Fig. 2 ). The first solution we thought of was mechanical lithotripsy, and as we feared, the wires broke near the handle of the lithotripter ( Fig. 3 a, b ). Attempted reduction of the calculi with forceps and re-cannulating the MPD both failed. We employed a DualKnife (KD-650U, Olympus Corporation, Tokyo, Japan) to extend the sphincterotomy by approximately 3 mm. With the exposure of the trapped pancreatolith, forceps removed part of the calculi at the papilla. A guidewire was then successfully passed through the fractured basket and pancreatolith complex, and the impacted complex was retrieved with a balloon (AMH-RBT; Anrei Medical, Hangzhou, China). Following this, the fractured basket was also successfully removed by forceps ( Video 1 ). The fragmented pancreatolith was also successfully removed by balloon and the patient was discharged uneventfully.

**Fig. 1:**
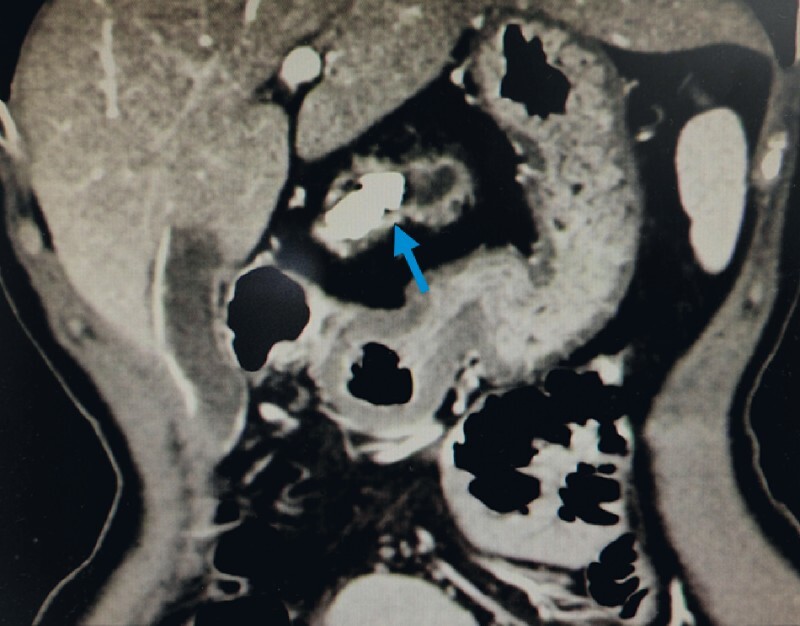
A radiopaque stone within the dilated pancreatic duct seen on computed tomography.

**Fig. 2:**
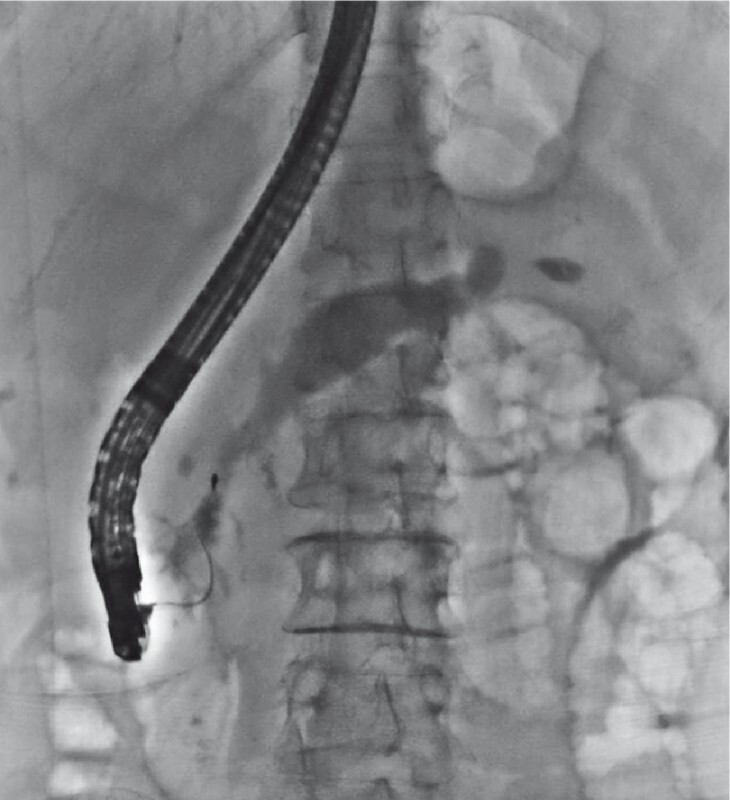
The basket with the entrapped stone was impacted within the pancreatic duct at the papilla.

**Fig. 3 a:**
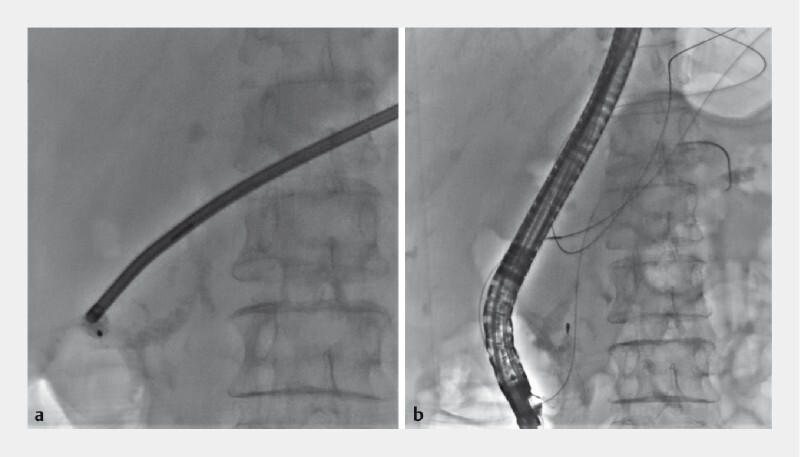
A captured pancreatolith with mechanical lithotripsy being performed. **b** The basket fractured at the handle portion of the lithotripter and the wires fell into the digestive tract.

**Video 1** Procedural steps of attempts to remove the trapped basket.


Here, we report a successful retrieval of an impacted basket with the entrapped pancreatolith. Although the situation may have been reported in the literature 1 2 3 , the operative procedure was thrilling and fascinating. We have shown that sphincterotomy extension is safe and effective in the management of this condition.

Endoscopy_UCTN_Code_CPL_1AK_2AF

## Notes

Endoscopy E-Videos

https://eref.thieme.de/e-videos
*E-Videos* is an open access online section of the journal *Endoscopy* , reporting on interesting cases and new techniques in gastroenterological endoscopy. All papers include a high-quality video and are published with a Creative Commons CC-BY license. Endoscopy E-Videos qualify for HINARI discounts and waivers and eligibility is automatically checked during the submission process. We grant 100% waivers to articles whose corresponding authors are based in Group A countries and 50% waivers to those who are based in Group B countries as classified by Research4Life (see: https://www.research4life.org/access/eligibility/ ). This section has its own submission website at https://mc.manuscriptcentral.com/e-videos

## References

[1] Thomas M Howell D A Carr-Locke D Mechanical lithotripsy of pancreatic and biliary stones: complications and available treatment options collected from expert centers Am J Gastroenterol 2007 102 1896 1902 17573790 10.1111/j.1572-0241.2007.01350.x

[2] Cho M K Song T J Park D H Extracorporeal shock wave lithotripsy allows successful endoscopic removal of a fractured stone basket trapped in the pancreatic duct Endoscopy 2016 48 E65 E66 26890546 10.1055/s-0042-101410

[3] Ryozawa S Iwano H Taba K Successful retrieval of an impacted mechanical lithotripsy basket: a case report Dig Endosc 2010 22 S111 S113 20590757 10.1111/j.1443-1661.2010.00963.x

